# The Hospital Saturday Committee

**Published:** 1922-10

**Authors:** F. Squires


### THE HOSPITAL SATURDAY COMMITTEE.

By F. SQUIRES.

ONE of the greatest individual sources of income of the voluntary hospital is that derived from workpeople's contributions. The organisation of these contributions is carried out by representatives of the workshops and other units of industry, and a number of *ex officio* members, who constitute the Hospital Saturday Committee. The aim of this committee is to obtain and maintain regular weekly contributions from every working man and woman in the district. When one realises that the " working classes " comprise the greater portion of the populace, it is readily seen that the achievement of the aforementioned aim would go far to solve the problem of the upkeep of the voluntary hospital. It is therefore vitally important that this Hospital Saturday Committee should be a " live " committee.

Sir James Michelli, in his address on " Thirty-Five Years of Hospital Life," states that a little over thirty years ago, " the hospital doors were closed against the general public," and that " mystery and superstition brooded over the hospitals." It is at about this period that Workpeople's Committees began to exist, and it is not surprising to find that, by reason of the " closed door," prejudice against the hospitals abounded, and still exists in a smaller degree to-day. The first step, then, is to obtain the confidence of the public by opening the doors of our institutions in every possible way. It should be borne in mind that not only do members of the Saturday Committee represent their fellow-employees, but *they also represent the hospitals in the workshop.*

Some of the following methods may be adopted in order to gain this confidence of the worker, necessary to the formation of a " live " Workpeople's Committee :—

The Committee should be represented on the Management Committees of the hospitals concerned, preferably by the Chairman and one or more members, who should report periodically to the Hospital Saturday Committee all matters dealt with by the Management Committees affecting the interests of the workpeople. Likewise, members of the Management Committees should be *ex officio* members of the Hospital Saturday Committee, and should regularly attend meetings of the latter body. The means will thus be provided for a regular interchange of ideas in connection with all matters relating to the management of the institution.

Members of the honorary medical staff should be asked to give popular lectures to the Committee on their particular branches of medicine or surgery. To these lectures all workers should be welcome. Nothing but good can result from such an arrangement.

Saturday afternoon visits to the hospitals by parties from the various works should be arranged. The result of practical insight into the work of the institutions will be far-reaching.

It is a good plan occasionally to combine the business of the Committee with a little pleasure. If the hospitals possess convalescent homes in the country within easy access, visits should be organised in the summer months, and members and friends invited. The small cost of these excursions is amply repaid by the increased interest created in the provision made for the convalescent patient. In the winter smoking concerts could be arranged, during the progress of which interesting discussions could take place on hospital matters.

Meetings of the Hospital Saturday Committee should be held regularly, whether or not any particular business is for discussion. When the interest of the worker has once been aroused in the work of the institution, a good attendance and a successful meeting will always be assured.

It is a good plan for members of the Committee to have official badges issued to them during their term of office, so that they will become known to their fellow-employees as the hospitals' representatives. Fellow-workers should be encouraged to bring to their notice any matter occurring in the workshop affecting the hospitals, the representative providing the means of intercourse between the worker and the hospitals.

At a Midland hospital, situated in an industrial area, where the above methods have been adopted, the workpeople's contributions for the year 1921, despite the great amount of unemployment which existed during the period, reached a sum within 7½ per cent. of the amount collected in 1920.

